# Increased hippocampal activity in children with autism after transcranial electrical stimulation

**DOI:** 10.1192/j.eurpsy.2026.11742

**Published:** 2026-07-31

**Authors:** C. J. Vera Scamarone

**Affiliations:** Neuroscience, OMMURA, Lima, Peru

## Abstract

**Introduction:**

Transcranial electrical stimulation (tDCS) is a no-invasive brain stimulation technique that had may usefully in mental health around the world, specially in Europe. Applied in autism and AHAD has improve in hyperactivity symptoms. Anodal stimulation on the left dorsolateral prefrontal cortex was the most chosen form of use in autism. Now we value the activation of areas with hypofunction and see the results.

**Objectives:**

Cuantification of the improve with brain mapping images in autism, correlationship with phenomenology symptoms.

**Methods:**

Brain mapping is performed using the KitWawe Amplifier 2000, and each patient undergoes two brain mappings: one at the beginning and one at the end of the 20 sessions.

Transcranial electrical stimulation was performed with Plato Work devices from Denmark, using the 2-mA front-occipital montage, anode-cathode.

Patients with Autism Spectrum Disorder type 1 ranged in age from 6 to 14 years, with a mean age of 9 years, and were predominantly male.

**Results:**

The population sample included males and females between 7 and 17 years of age, forming a unitary sample of 10 males and 10 females, with the standard average age being 11 years and 6 months.To measure the variation in the connection between the frontal and occipital, 3D brain mapping images were used from the first stimulation session and in the twentieth session. The images show an increase in detailed connectivity between the left frontal and occipital regions in 71% of patients, and a decrease in left disjunction in 10%. Only 19% of patients showed no changes in brain mapping, which are inconsistent with clinical improvement.

**Conclusions:**

We observed that 81 % of patients had increased frontal-occipital connectivity with increased signals from the midbrain, as well as decreased activity in the left amygdala. Furthermore, behavior improved in 93 % of patients, with decreased impulsivity, mannerisms, and stereotypies, and improved verbal response and calm.

The measurement of midbrain signals associated with autism spectrum disorder is quantifiable with 3D imaging as well as clinically. The results are maintained despite a six-month follow-up. Further studies are recommended, as well as the validation of transcranial electrical stimulation as an effective method for the treatment of autism.

**Disclosure of Interest:**

None Declared

